# Gene evolution and gene expression after whole genome duplication in fish: the PhyloFish database

**DOI:** 10.1186/s12864-016-2709-z

**Published:** 2016-05-18

**Authors:** Jeremy Pasquier, Cédric Cabau, Thaovi Nguyen, Elodie Jouanno, Dany Severac, Ingo Braasch, Laurent Journot, Pierre Pontarotti, Christophe Klopp, John H. Postlethwait, Yann Guiguen, Julien Bobe

**Affiliations:** INRA, Laboratoire de Physiologie et Génomique des poissons, Campus de Beaulieu, F-35042 Rennes cedex, France; INRA, SIGENAE, GenPhySE, F-31326 Castanet-Tolosan, France; INRA, SIGENAE, UR 875, MIAT INRA, Toulouse, France; CNRS, MGX-Montpellier GenomiX, Montpellier, France; Aix-Marseille Université, CNRS, Centrale Marseille, I2M, UMR7373, FR 4213 - FR, Eccorev 3098, équipe EBM, 13331 Marseille, France; Institute of Neuroscience, University of Oregon, Eugene, 97403-1254 OR USA; Department of Integrative Biology, Michigan State University, East Lansing, 48824 MI USA

**Keywords:** Gene duplication, Teleosts, Holostean, Gene expression, Gar, Salmonids, Assembly, Stra8, Mcam

## Abstract

With more than 30,000 species, ray-finned fish represent approximately half of vertebrates. The evolution of ray-finned fish was impacted by several whole genome duplication (WGD) events including a teleost-specific WGD event (TGD) that occurred at the root of the teleost lineage about 350 million years ago (Mya) and more recent WGD events in salmonids, carps, suckers and others. In plants and animals, WGD events are associated with adaptive radiations and evolutionary innovations. WGD-spurred innovation may be especially relevant in the case of teleost fish, which colonized a wide diversity of habitats on earth, including many extreme environments. Fish biodiversity, the use of fish models for human medicine and ecological studies, and the importance of fish in human nutrition, fuel an important need for the characterization of gene expression repertoires and corresponding evolutionary histories of ray-finned fish genes. To this aim, we performed transcriptome analyses and developed the PhyloFish database to provide (i) *de novo* assembled gene repertoires in 23 different ray-finned fish species including two holosteans (i.e. a group that diverged from teleosts before TGD) and 21 teleosts (including six salmonids), and (ii) gene expression levels in ten different tissues and organs (and embryos for many) in the same species. This resource was generated using a common deep RNA sequencing protocol to obtain the most exhaustive gene repertoire possible in each species that allows between-species comparisons to study the evolution of gene expression in different lineages. The PhyloFish database described here can be accessed and searched using RNAbrowse, a simple and efficient solution to give access to RNA-seq *de novo* assembled transcripts.

## Background

Ray-finned fish occupy a wide diversity of aquatic habitats. More than 30,000 ray-finned fish (Actinopterygii) species have been reported that account for approximately half of vertebrates on earth [1]. A vast majority of ray-finned fish are teleosts with only 50 non-teleost species reported. Ray-finned fish evolution spanned more than 400 million years [2–4]. In addition to the two rounds of whole genome duplications that occurred at the root of the vertebrate lineage (VGD1 and VGD2) [5], teleost fish experienced a third round of WGD [6–8]. This teleost-specific round of WGD (TGD) occurred 320–350 million years ago (Mya), after the divergence between the holostean lineage, which includes Semionotiformes (gars) and Amiiformes (bowfin), and the lineage leading to teleost [9, 10]. Additional WGD events have also been described in the teleost lineage [11, 12], including the salmonid-specific WGD (SaGD) that occurred about 100 Mya [13, 14].

After duplication, the most likely fate of duplicated genes is the loss of one of the duplicates through non-functionalization (also known as pseudogenization) that occurs by accumulation of deleterious mutations [15–17]. While common after WGD, gene loss could however play a key role in speciation [18], through a process known as divergent resolution [19]. In addition, duplicated genes may also be retained in two copies and either specialize by the partitioning of ancestral gene functions (i.e. subfunctionalization) or by the acquisition of a novel function (i.e. neofunctionalization) [20]. In rainbow trout (*Oncorhynchus mykiss*), 100 million years after WGD (i.e. SaGD), 48 % of the ancestral genes have been retained in duplicates, while 52 % have resorted to singletons. Among duplicated gene pairs originating from WGD, which are also called ohnologs [21], differences are observed in the expression patterns and levels of the two copies, as shown in rainbow trout [13].

Analysis of gene expression in teleosts is therefore made interesting by the huge diversity of species (>30,000), lineage-specific gene losses, differential sub- and neofunctionalization, and additional rounds of WGD found in this group. In addition, high quality genomic resources (i.e. fully assembled genome) remain scarce, despite a recent and substantial increase in the number of sequenced genomes publicly available. Existing fish genome resources however still lack many important nodes in teleost diversity and evolution and, for instance, more than 80 % of species with sequenced genomes lie within the Euteleostei lineage, leaving out many basally diverging lineages. In line with that lack of an evolutionary based dataset of teleost genomes, our knowledge of expressed gene repertoires remains also extremely limited and skewed towards specific branches within the teleost tree of life. Significant resources exist in some lineages (e.g. percomorphs) while they are scarce in other lineages (e.g. osteoglossomorphs). The lack of data generated using similar (or at least comparable) methodologies across several species that make comparative analysis possible is a hurdle for understanding.

For reasons listed above, it is currently difficult to compare gene expression among teleost species due to (i) the lack of an exhaustive gene repertoire in all but a few species and (ii) the lack of expression data collected using comparable methodologies across the same tissues and stages in different species. The PhyloFish database was designed to address both questions and provides a comprehensive gene repertoire from *de novo* assembled RNA-seq data in a large number of species chosen to entirely span the ray-finned fish tree of life with special attention for TGD and SaGD WGD events. The PhyloFish database also provides consistent gene expression data in the same tissues and organs in the different species to allow between-species comparisons. The PhyloFish database is therefore a unique and essential resource to study the evolution of gene expression in the different ray-finned fish lineages that will be extremely useful in many biological fields such as ‘evo-devo’, ecology, toxicology, aquaculture, and physiology.

## Construction and content

### Species selection and tissue collection

**Fig. 1:**
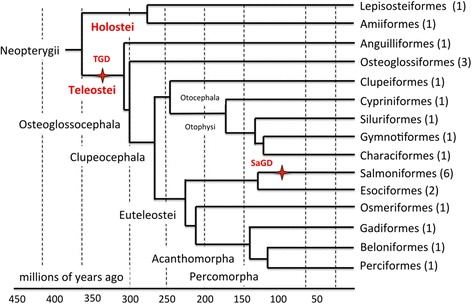
Phylogenetic tree of the PhyloFish species. Cladogram showing phylogenetic relationships among ray-finned fish analyzed in the present study. Tree topology was adapted from [2]. For each phylogenetic group, the number of species in the PhyloFish set is indicated between brackets. The teleost specific (TGD) and salmonid-specific (SaGD) whole genome duplication events are indicated in red

Fish used in this study were reared and handled in strict accordance with French and European policies and guidelines of the INRA LPGP Institutional Animal Care and Use Committee (# 25 M10), which approved this study. Species included in the PhyloFish database (Fig. 1, Table 1) were chosen not only for their evolutionary position in the tree of life [2, 22] but also, when possible, for their ecological and economical relevance. A total of 23 species were included in the database to span two different whole genome duplication events found in teleost fish. Different species were therefore selected before the TGD (Holosteans, *N* = 2 species), after the TGD and before the SaGD (TGD teleosts, *N* = 15 species), and after SaGD (SaGD teleost, *N* = 6 species). Bowfin (*Amia calva*) and spotted gar (*Lepisosteus oculatus*) were selected among holosteans. Because the holostean lineage diverged from teleosts before the teleost-specific third round of whole genome (TGD) duplication, they provide useful information on the gene repertoire before the TGD and serve as an outgroup to evaluate gene evolution after the TGD. To provide a global view of gene expression patterns in TGD teleosts, 15 species were selected among the following groups: Anguilliformes, Osteoglossiformes, Clupeiformes, Cyprinformes, Siluriformes, Gymnotyformes, Characiformes, Esociformes, Osmeriformes, Gadiformes, Beloniformes, and Perciformes. While a single species was selected in most groups, three species (butterfly fish, Arowana, and elephantnose fish) were selected in the Osteoglossiformes because they diverged shortly after TGD and have few available transcriptomic resources. In addition, two species (Pike (*Esox lucius*) and Eastern mudminnow (*Umbra pygmaea*)) were selected among Esociformes as this group serves as the most closely related outgroup to study evolution after the SaGD. After SaGD, six species were selected among Salmoniformes, providing a unique opportunity to explore the evolution of gene expression and function after a comparatively recent animal genome duplication event.

**Table 1:** Species present in the PhyloFish database

Name	Species	Phylogenetic group	Nb of contigs	WGD
Bowfin	*Amia calva*	Amiiformes	35064	VGD2
Spotted gar	*Lepisosteus oculatus*	Lepisosteiformes	41396	VGD2
European eel	*Anguilla anguilla*	Anguilliformes	60263	TGD
Butterfly fish	*Pantodon buchlolzi*	Osteoglossiformes	44577	TGD
Arowana	*Osteoglossum bicirrhosum*	Osteoglossiformes	55739	TGD
Elephantnose fish	*Ghnathonemus petersi*	Osteoglossiformes	53423	TGD
Aliss shad	*Alosa alosa*	Clupeiformes	53363	TGD
Zebrafish	*Danio rerio*	Cypriniformes	48158	TGD
Panga	*Pangasius hypophthalmus*	Siluriformes	43013	TGD
Black ghost knifefish	*Apteronotus albifrons*	Gymnotiformes	45356	TGD
Mexican tetra (cave)	*Astyanax mexicanus*	Chraraciformes	47729	TGD
Mexican tetra (surface)	*Astyanax mexicanus*	Characiformes	46670	TGD
Northern pike	*Esox lucius*	Esociformes	48567	TGD
Eastern mudminnow	*Umbra pygmae*	Esociformes	46381	TGD
Grayling	*Thymallus thymallus*	Salmoniformes	67157	SaGD
European whitefish	*Coregonus lavaretus*	Salmoniformes	74701	SaGD
American whitefish	*Coregonus clupeaformis*	Salmoniformes	66996	SaGD
Brown trout	*Salmo trutta*	Salmoniformes	75338	SaGD
Rainbow trout	*Oncorhynchus mykiss*	Salmoniformes	78415	SaGD
Brook trout	*Salvelinus fontinalis*	Salmoniformes	69441	SaGD
Sweetfish	*Pecoglossus altivelis*	Osmeriformes	47484	TGD
Atlantic cod	*Gadus morhua*	Gadiformes	50564	TGD
Medaka	*Orysias latipes*	Beloniformes	42186	TGD
European perch	*Perca fluviatilis*	Perciformes	49204	TGD

For each species, the common name (according to fishbase.org), the species name, phylogenetic group, the number of *de novo* assembled contigs generated, and position related to successive whole genome duplication (WGD) are shown. VGD2 (vertebrate 2^nd^ round of WGD), TGD (teleost-specific WGD), SaGD (salmonid specific WGD)

For each species included in the database we constructed separate libraries from the following tissues or organs to allow analysis of tissue specific expression patterns: brain, liver, gills, heart, muscle, liver, kidney, bones, intestine, ovary, and testis. The gill library for blackghost knifefish (*Apteronotus albifrons*) is lacking due to limiting RNA quality libraries for embryos or larvae were made for gar (*Lepisosteus oculatus*), European eel (*Anguilla anguilla*), Aliss shad (*Alosa alosa*), zebrafish (*Danio rerio*), panga (*Pangasius hypophthalmus*), Northern pike (*Esox lucius*), grayling (*Thymallus thymallus*), Atlantic cod (*Gadus morhua*), medaka (*Orysias latipes*), Eurasian perch (*Perca fluviatilis*), brook trout (*Salvelinus fontinalis*), Mexican tetra (*Astyanax mexicanus*, both cave and surface populations), and European whitefish (*Coregonus lavaretus*). For all species, tissues were sampled from the same female individual and testis from a male individual, when possible. For rainbow trout, existing RNA-seq data previously used in the rainbow trout genome sequencing project were used [13]. In this study, tissues had been sampled from a gynogenetic female and the testis is missing. In some species and depending on the tissues, RNA samples from different individuals were pooled to obtain sufficient amounts of RNA for sequencing. All corresponding information is available in the biosample and bioproject files deposited in SRA under the PhyloFish umbrella project.

### RNA-seq

Sequencing libraries were prepared using a TruSeq RNA sample preparation kit, according to manufacturer instructions (Illumina, San Diego, CA). Poly-A-containing mRNA was isolated from total RNA using poly-T oligo-attached magnetic beads, and chemically fragmented. First-strand cDNA was generated using SuperScript II reverse transcriptase and random primers. Following the second strand cDNA synthesis and adaptor ligation, cDNA fragments were amplified by PCR. Products were loaded onto an Illumina HiSeq2000 instrument and subjected to multiplexed paired-end (2 × 100 bp) sequencing. The processing of fluorescent images into sequences, base-calling and quality value calculations were performed using the Illumina data processing pipeline.

### *de novo* transcriptome assembly

For each library, raw sequence data in fastq format were quality checked, stored in the ng6 database [http://www.biomedcentral.com/1471-2164/13/462], and filtered to remove unknown nucleotides. The longest subsequences without Ns exceeding half of the total read length were retained. Velvet and Oases performed transcriptome *de novo* assembly [23]. We first constructed nine independent assemblies for each library using different k-mers (k-mers for velveth: 25,31,37,43,49,55,61,65,69; parameters for velvetg: −read_trkg yes -min_contig_lgth 100 -cov_cutoff 4; parameters for oases: −cov_cutoff 4). Raw transcripts.fa files were filtered to retain only one transcript per locus based on the highest fold coverage using a Python script developed by a bioinformatic team at the Brown University (https://sites.google.com/a/brown.edu/bioinformatics-in-biomed/velvet-and-oases-transcriptome). Antisense chimeras accidentally produced during the assembly step were removed using a homemade script. Independent assemblies were pooled and duplicate/similar transcripts built by close k-mers were removed by a cd-hit-est [24] step (parameters: −M 0 -d 0 -c 0.98) and merged by a TGICL [25] step (parameters: −l 60 -p 96 -s 100000). After this assembly process, all input reads were mapped back to the set of transcripts using BWA [26] and the size of the longest open reading frames (ORFs) for each transcript was computed using the getorf EMBOSS tool [27]. Finally, transcripts were filtered using mapping rate and ORF length criteria. Transcripts with ORFs shorter than 200 nt and with fewer than two mapped reads per million of overall mapped reads were discarded. The above procedure was carried out independently for each tissue-specific library.

For each species, the library-specific assembly was followed by a meta-assembly step. The purpose of this step was to limit redundancy (i.e. identical transcript originating from different tissue libraries) in the final species-specific assembly. For each species, *de novo* assembled transcripts originating from the different tissue-specific libraries were pooled. The longest ORF of each transcript was extracted and ORFs were clustered using cd-hit (parameters: −M 0 -d 0 -c 0.90 -g 1). From each cd-hit cluster, the transcript with the longest ORF or the longest transcript (if more than one transcript had an ORF of the maximum size) was selected in order to increase the probability of retaining contigs with full-length coding sequence. Input reads from all conditions were mapped back to selected transcripts using BWA. Again, transcripts were filtered based on the re-mapping rate. Transcripts with less than one mapped read per million of overall mapped reads were discarded. Finally, it should be stressed that the longest ORF was not used for annotation, because annotation was performed for each retained transcript using a BlastX procedure against existing public databases.

### Transcriptome coverage and quality control

The *de novo* assembly procedure was trained and optimized using zebrafish, for which a genome-based high quality transcriptome is available. Our *de novo* assembly procedure yielded 48,158 contigs in zebrafish. This number is consistent with the 25,642 coding gene and 57,369 gene transcripts predicted from the latest Ensembl genome assembly (GRCz10, 2014). The number of PhyloFish contigs for zebrafish is lower that the total number of Ensembl zebrafish transcripts. This difference can be explained, at least in part, by the biological material used here (10 tissues, each being sampled at a single biological stage) that does not cover all possible biological conditions. The number of transcript contigs scaled with the number of WGD events, from holeosteans (two) to most teleosts (three) to salmonids (four) (Table 1). While the lowest number of contigs (<41,500, *N* = 2) was found in holosteans, it ranged between 42,200 and 60,200 in TGD teleosts (*N* = 15) and between 67,000 and 78,400 in SaGD species (*N* = 6). These figures track with the number of genes resulting from the different WGD events found in the analyzed species. In rainbow trout, a SaGD species, it has been shown that 48 % of the duplicated genes originating from SaGD were retained in two copies [13]. The mean number of contigs in the 15 TGD but non-SaGD species present in the PhyloFish database was 48,900, while it was 72,000 for the six SaGD salmonid species, on average. We have therefore generated 47 % more contigs in SaGD species in comparison to TGD species, in agreement with the percent of duplicated gene retention after WGD in salmonids (i.e. 48 %). It should also be noted that the number of contigs was strikingly similar in the two populations of Mexican tetra that diverged recently and are therefore likely to exhibit a similar number of genes and transcripts (46,670 and 47,729 contigs were generated in surface and cave populations, respectively). Finally, when training the assembly using the zebrafish genome, we calculated that more than 75 % of zebrafish contigs aligned to the zebrafish protein repertoire using BLAT with >80 % identity and >80 % coverage of the overall protein length, further validating assembly methodologies.

Together, these results indicate that the number of contigs in each species is consistent with the number of existing genes and transcripts, and that transcriptome coverage is also substantial in terms of both number of proteins and overall protein coverage despite using just 10 tissues and only one developmental timepoint.

For all species, contigs were aligned using blast against the refseq_protein and swissprot protein databases (blastx -e 1e-5 -F T -v 20 -b 20) as well as several nucleic acid databases, including Unigene *Danio rerio* version 126, *Oryzias latipes* 130 and Ensembl 71 transcripts of *Danio rerio*, *Oryzias latipes*, *Takifugu rubripes* and *Tetraodon nigroviridis* and RefSeq_RNAs (June 2013). The GO (gene ontology) annotations of aligned proteins were retrieved and stored in the database.

## Utility and discussion

### Database features

The PhyloFish database is made available (http://phylofish.sigenae.org/index.html) through the internet using RNAbrowse, which provides a simple and efficient access to RNA-Seq *de novo* assembled transcripts [28]. RNAbrowse offers many features that will help users analyze and extract biologically meaningful information from the PhyloFish data. The PhyloFish web browser offers several different possible modes of analysis. For each species, which can be selected in the front page by a simple click on the species name, an overview is provided that includes a set of graphics showing general statistics, containing for example the contig length histogram. The browser also includes detailed information about the different sequenced libraries and provides access to tools such as Venn diagrams and digital differential display. A blast query form is available to align a known sequence on all contigs. The query must be provided using a fasta or multi fasta format. The search can also be done using a name or description through the biomart form. Users can then add retrieved contigs to the favorite table. For each contig, the sequence can be extracted to perform a multiple alignment to check if different splice forms have been assembled. All possible open reading frames can be visualized and annotations can be graphically displayed using jbrowse [29]. It is also possible to graphically visualize expression levels along the contigs in the different libraries. Expression data in the various libraries can be exported to generate expression profiles for the different tissues/organs. To our knowledge, the PhyloFish database is the only database that allows (i) the identification of contigs in such a large diversity of fish species, including many species with no or limited transcriptomic resources, and (ii) the generation of tissue expression patterns from 23 different species (including two holosteans) in which the same tissues were sampled by consistent methodologies and for which the RNA-seq procedure is similar (i.e. with the same chemistry, the same type of library, and the same sequencing depth), all features that promote normalized comparisons across tissues and taxa.

### Case study

**Fig. 2:**
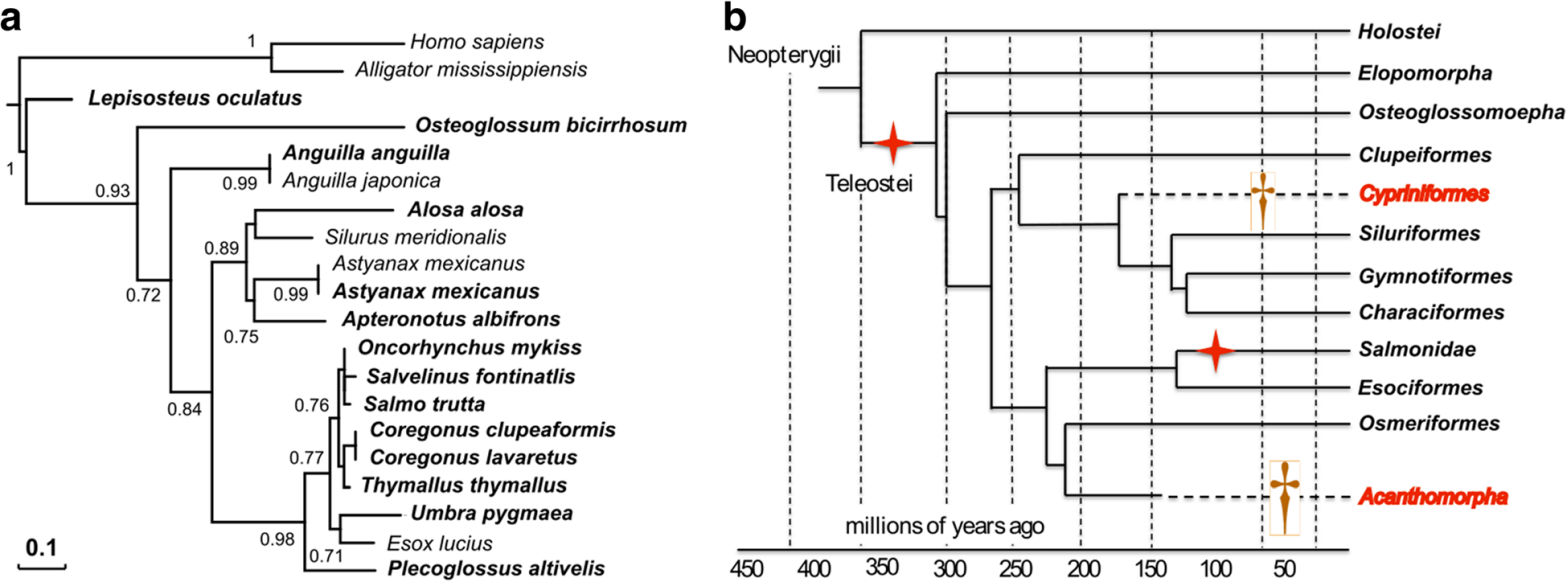
Stra8 proposed gene evolution in teleosts following the TGD WGD. Maximum-likelihood phylogeny of Stra8 (**a**) was performed using the PhyML software [38] implemented in the Phylogeny.fr web platform [39] using default “a la carte” parameters and a bootstrapping procedure (*N* = 100 bootstraps). The resulting tree was exported and edited in Evolview [40]. Input sequences were retrieved using a tblastn search of the PhyloFish database using as bait the Southern catfish Stra8 protein (AGM53488.1). PhyloFish Stra8 coding sequences (in bold on the tree) were submitted to GenBank with the following accession numbers: *Lepisosteus oculatus* (KU161162), *Osteoglossum bicirhosum* (KU161164), *Anguilla anguilla* (KU161163), *Alosa alosa* (KU161165), *Astyanax mexicanus* (KU161166), *Apteronotus albifrons* (KU161167), *Oncorhynchus mykiss* (KU161168), *Salvelinus fontinalis* (KU161169), *Coregonus lavaretus* (KU161172), *Coregonus clupeaformis* (KU161171), *Salmo trutta* (KU161170), *Thymallus thymallus* (KU161173), *Umbra pygmae* (KU161174) and *Plecoglossus altivelis* (KU161175). This dataset was complemented with two additional teleost public sequences of Stra8 in *Esox lucius* (XP_012986862) and *Astyanax mexicanus* (XP_007229918.1) and a Stra8 sequence deduced from the *Anguilla japonica* genome (scaffold 6093). The tree was rooted with tetrapod sequences using the *Homo sapiens* STRA8 (AAP47163.1) and *Alligator mississippiensis* Stra8 (XP_006261218.1). **b** Schematic representation of the deduced evolution of *stra8* based on PhyloFish sequences. This analysis suggests that *stra8* was completely lost in Acanthomorpha, but also specifically and independently lost in the Cypriniformes lineage. The tree is based on [2]

**Fig. 3:**
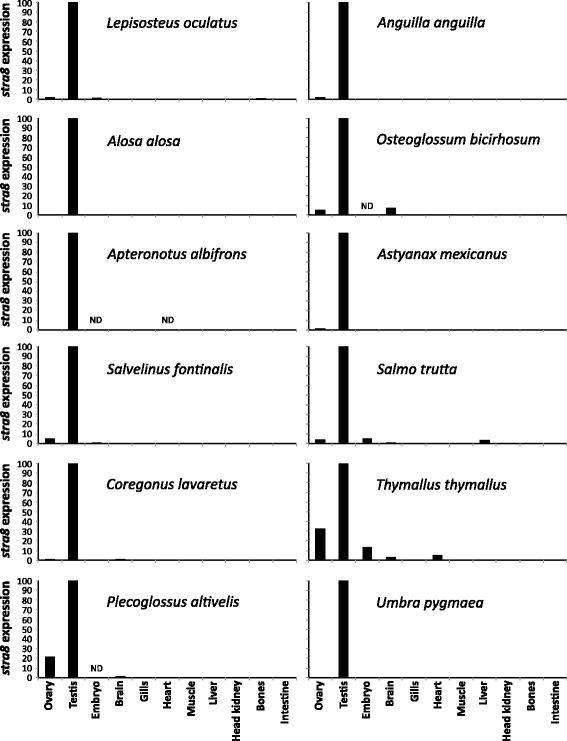
Tissue expression profiles of *stra8* reveal expression predominantly in testes in most PhyloFish species. Relative expression of *stra8* was calculated as the percentage of the maximum rpkm (number of reads per kilobase per million reads) value per species. ND: no data (tissue not sequenced in that species)

To illustrate the utility of the PhyloFish database to solve problems of gene evolutionary history, we used it to decipher the evolutionary history of *stra8* (Stimulated by retinoic acid gene 8), and subsequently characterize its expression in holosteans and teleosts. The *stra8* gene encodes a retinoic acid-responsive protein that is involved in the regulation of meiotic initiation during spermatogenesis and oogenesis [30]. This gene was first hypothesized to be lost either in the ray-finned fish lineage or in the teleost lineage [31]. This assumption was mainly based on its absence from the zebrafish genome and other teleost reference genomes available at that time (i.e. stickleback, Tetraodon, fugu, medaka). The loss of *stra8* in teleosts was however recently challenged by the discovery of an apparent *stra8* ortholog (AGM53488.1) in Southern catfish (*Silurus meridionalis*) [32]. We revisited *stra8* gene evolution using the PhyloFish database as a main resource. Using the Stra8 protein sequence from Southern catfish [32], we queried PhyloFish databases and retrieved fourteen sequences with a significant Stra8 hit in thirteen teleosts and one holostean species (species in bold type in Fig. 2a). These fourteen sequences were used in a phylogenetic analysis combined with the Southern catfish Stra8 protein sequence used as bait in our analysis and three additional teleost Stra8 sequences available in public databases (*Esox lucius*, *Astyanax mexicanus*, and *Anguilla japonica*). Phylogenetic analysis revealed that all these PhyloFish sequences are true orthologs of the tetrapods and the southern catfish *stra8* gene (Fig. 2a) and that only a single *stra8* paralog was retained after the TGD whole genome duplication. No *stra8* sequence was found in the PhyloFish database for zebrafish, cod, medaka, and European perch, thus corroborating previous reports based on zebrafish and Acanthomorpha genome analysis [31]. No *stra8* homolog was detected in public databases in any Cypriniform species (e.g., carps) even after an extensive search of GenBank nucleotide collection (nr/nt), Expressed Sequence Tags (ESTs), Transcriptome Shotgun Assembly (TSA), and NCBI genomes. This surprising finding strongly suggests that *stra8* was lost in Acanthomorpha (Fig. 2b), and independently lost in the Cypriniform lineage. In addition, using the PhyloFish database, we explored the tissue expression of *stra8* genes, showing that *stra8* is mainly gonadal with a predominant expression in the testis (Fig. 3), as previously shown in the Southern catfish [32] and in mammals [33].

**Fig. 4:**
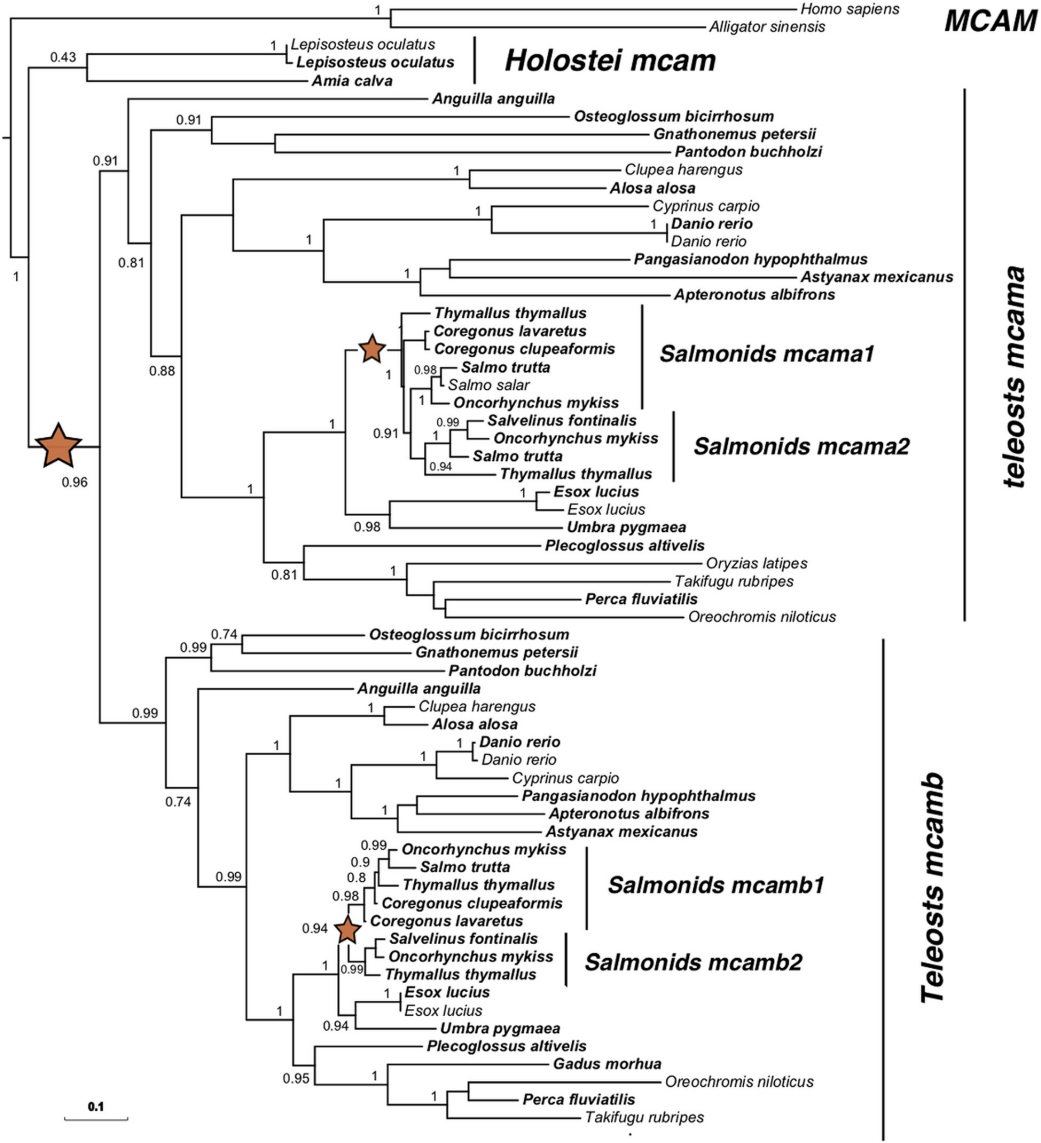
Phylogeny of Mcam in teleosts following the TGD and SaGD WGDs. Maximum-likelihood phylogeny of Mcam was performed using the PhyML software [38] implemented in the Phylogeny.fr web platform [39] using default “a la carte” parameters and a bootstrapping procedure (*N* = 100 bootstraps). The resulting tree was exported and edited in Evolview [40]. Input sequences were retrieved using a tblastn search of the PhyloFish database using as bait the zebrafish Mcam protein (XP_005157627.1), in the Mcama branch of the tree. PhyloFish Mcam coding sequences are shown in bold on the tree. The tree was rooted with tetrapod sequences using the Homo sapiens MCAM (AAH56418) and Alligator sinensis Mcam (XP_014373905). A few additional published teleosts Mcam sequences were added in the analysis (normal font). The TGD and SaGD are shown with red stars

In addition to the *stra8* case study presented above that highlights a very specific case of evolution after duplication (i.e., loss of one copy of a duplicated pair of paralogs and lineage-specific losses of the second copy) we also investigated a more classical case of gene evolution. We thus characterized the evolutionary history of *mcam* (*melanoma cell adhesion molecule*) (also known as *cd146*). This gene encodes a protein with known roles in cell adhesion and in cohesion of the endothelial monolayer at intercellular junctions in vascular tissue [34, 35]. Using a combination of sequences originating from sequences available in GenBank and from the PhyloFish database, we reconstructed the evolutionary history of the *mcam* gene (Fig. 4). This gene was retained as two paralogous copies after the TGD with an additional complete retention of duplicated paralogs in the salmonid lineage after the SaGD. This gene follows a complete 1 (Tetrapods and Holostei) to 2 (Teleosts) to 4 (Salmonids) duplication rule with a total retention of paralogs after two round of whole genome duplication leading to four copies in salmonids.

PhyloFish data were also used to characterize the evolution of the expression of *prrx1* and *prrx2* genes, two VGD ohnologs, in teleosts compared to the spotted Gar. We concluded that for *prrx*, the spotted gar genome and gar gene expression patterns mimic mammals better than teleosts do, and that there is significant diversity among teleost lineages with respect to the loss and retention of *prrx* TGD ohnologs [36]. Finally, the PhyloFish database was recently used by the Spotted Gar Genome Consortium to thoroughly analyze the evolution of gene expression after TGD using spotted gar, zebrafish, and medaka [37].

## Conclusions

The PhyloFish database is a unique resource providing comprehensive expressed gene repertoires collected and processed using the same protocol for 23 ray-finned fish species. This resource is currently the only database offering the possibility to analyze gene expression after genome duplication in teleost fish, including salmonids, in such a comprehensive and comparative way. The PhyloFish database has already proved its utility and will be of further interest in many biological fields such as ‘evo-devo’, ecology, toxicology, aquaculture, and physiology. In the future, the PhyloFish database can be expanded to incorporate data from other fish species to broaden its scope and explore gene evolution in many different teleost lineages.

## Availability and requirements

The PhyloFish database is available online at http://phylofish.sigenae.org/index.html. All sequences described in this paper can be downloaded from that site. RNA-seq raw sequence data from the Hiseq2000 sequencer have been deposited into the NCBI SRA under accessions SRP044781 (zebrafish), SRP044782 (spotted gar), SRP044783 (bowfin), SRP044784 (medaka), SRP045098 (black ghost knifefish), SRP045099 (European eel), SRP045100 (butterfly fish), SRP045101 (brown trout), SRP045102 (arowana), SRP045103 (aliss shad), SRP045138 (eastern mudminnow), SRP045139 (rainbow trout), SRP045140 (panga), SRP045141 (northern pike), SRP045142 (grayling), SRP045143 (European whitefish), SRP045144 (European perch), SRP045145 (elephantnose fish), SRP045146 (sweetfish), SRP058861 (lake whitefish), SRP058862 (brook trout), SRP058863 (cave fish), SRP058865 (Atlantic cod), and SRP058863 (surface fish).
